# Serine protease HtrA1 accumulates in corneal transforming growth factor beta induced protein (TGFBIp) amyloid deposits

**Published:** 2013-04-12

**Authors:** Henrik Karring, Ebbe Toftgaard Poulsen, Kasper Runager, Ida B. Thøgersen, Gordon K. Klintworth, Peter Højrup, Jan J. Enghild

**Affiliations:** 1 Institute of Chemical Engineering, Biotechnology and Environmental Technology, Faculty of Engineering, University of Southern Denmark, Odense, Denmark; 2 Department of Molecular Biology and Genetics, Science Park, Aarhus University, Aarhus, Denmark; 3 Departments of Pathology and Ophthalmology, Duke University Medical Center, Durham, NC; 4 Department of Biochemistry and Molecular Biology, University of Southern Denmark, Odense, Denmark

## Abstract

**Purpose:**

Specific mutations in the transforming growth factor beta induced (*TGFBI*) gene are associated with lattice corneal dystrophy (LCD) type 1 and its variants. In this study, we performed an in-depth proteomic analysis of human corneal amyloid deposits associated with the heterozygous A546D mutation in *TGFBI*.

**Methods:**

Corneal amyloid deposits and the surrounding corneal stroma were procured by laser capture microdissection from a patient with an A546D mutation in *TGFBI*. Proteins in the captured corneal samples and healthy corneal stroma were identified with liquid chromatography-tandem mass spectrometry and quantified by calculating exponentially modified Protein Abundance Index values. Mass spectrometry data were further compared for identifying enriched regions of transforming growth factor beta induced protein (TGFBIp/keratoepithelin/βig-h3) and detecting proteolytic cleavage sites in TGFBIp.

**Results:**

A C-terminal fragment of TGFBIp containing residues Y571-R588 derived from the fourth fasciclin 1 domain (FAS1–4), serum amyloid P-component, apolipoprotein A-IV, clusterin, and serine protease HtrA1 were significantly enriched in the amyloid deposits compared to the healthy cornea. The proteolytic cleavage sites in TGFBIp from the diseased cornea are in accordance with the activity of serine protease HtrA1. We also identified small amounts of the serine protease kallikrein-14 in the amyloid deposits.

**Conclusions:**

Corneal amyloid caused by the A546D mutation in *TGFBI* involves several proteins associated with other varieties of amyloidosis. The proteomic data suggest that the sequence 571-YHIGDEILVSGGIGALVR-588 contains the amyloid core of the FAS1–4 domain of TGFBIp and point at serine protease HtrA1 as the most likely candidate responsible for the proteolytic processing of amyloidogenic and aggregated TGFBIp, which explains the accumulation of HtrA1 in the amyloid deposits. With relevance to identifying serine proteases, we also found glia-derived nexin (protease-nexin 1) in the amyloid deposits, making this serine protease inhibitor a good candidate for the physiologically relevant inhibitor of one of the amyloid-associated serine proteases in the cornea and probably in other tissues. Noteworthy, the present results are in accordance with our findings from a previous study of corneal amyloid deposits caused by the V624M mutation in *TGFBI*, suggesting a common mechanism for lattice corneal dystrophies (LCDs) associated with mutations in the TGFBIp FAS1–4 domain.

## Introduction

Mutations in the transforming growth factor beta induced (*TGFBI*) gene encoding transforming growth factor beta induced protein (TGFBIp/keratoepithelin/βig-h3) are associated with several inherited corneal diseases with different clinical and histopathological characteristics [1]. The diseases include different phenotypes of lattice corneal dystrophy (LCD) type 1, granular corneal dystrophy (GCD), and Thiel-Behnke corneal dystrophy (TBCD) characterized by corneal deposition of amyloid, granular non-amyloid aggregates, and “curly fibers,” respectively [2]. It has become apparent that specific *TGFBI* mutations cause specific corneal phenotypes, but the molecular mechanism is not clear. However, our previous results show that the types of protein deposition (amyloid, granular, or “curly fiber”) caused by mutations in the fourth fasciclin 1 (FAS1–4) domain in TGFBIp are linked to the thermodynamic stability of TGFBIp [3].

In a recent analysis of the composition and proteolytic processing of corneal amyloid deposits isolated from a patient with a variant of LCD type 1 caused by a homozygous V624M mutation in the FAS1–4 domain of TGFBIp [4], we found that a C-terminal fragment of TGFBIp containing residues Y571-R588, serum amyloid P-component, apolipoprotein E, apolipoprotein A-IV, and clusterin were specifically enriched in the amyloid aggregates. Thus, the findings suggested that the sequence 571-YHIGDEILVSGGIGALVR-588, which precedes the mutated valine (V624M) in the FAS1–4 domain of TGFBIp, contained the amyloidogenic sequence in this LCD type 1 variant [4]. Until now, whether the same proteins and C-terminal TGFBIp fragment accumulate in corneal amyloid deposits from other TGFBIp-linked LCDs has not been known.

In the present study, we determined the composition of the amyloid deposits from another patient with a variant of LCD type 1 caused by a different mutation in the TGFBIp FAS1–4 domain (A546D positioned before the Y571-R588 sequence). Originally, this particular LCD type 1 variant was designated polymorphic corneal amyloidosis because of specific clinicopathologic features [5]. Mass spectrometry-based label-free proteomic quantitation of the corneal amyloid deposits revealed that TGFBIp is the predominant protein in the corneal amyloid deposits. Comparative proteomic analyses of the amyloid deposits with a healthy corneal stroma suggest that a C-terminal fragment of TGFBIp containing residues Y571-R588, serum amyloid P-component, apolipoprotein A-IV, clusterin, and serine protease HtrA1 are abundant and significantly enriched in the amyloid deposits. Serum amyloid P-component, apolipoprotein A-IV, and clusterin were also enriched in the periamyloid corneal tissue (tissue not containing major amyloid deposits) from the diseased cornea, suggesting that this tissue contains misfolded protein and probably minor amounts of amyloid. Detailed proteomic analysis of the mass spectrometry data revealed numerous non-tryptic cleavages in TGFBIp especially from the amyloid deposits. In conclusion, the accumulation of serine protease HtrA1 in the amyloid lesions and identification of numerous proteolytic cleavage sites in TGFBIp consistent with HtrA1 activity strongly support our previous observations, suggesting that HtrA1 is involved in the proteolytic processing of amyloidogenic TGFBIp in the cornea [4].

## Methods

### Preparation of corneal samples

Corneal samples were obtained from a 74-year-old female Caucasian patient with an LCD type 1 variant (previously designated polymorphic corneal amyloidosis) and a heterozygous A546D mutation in *TGFBI.* The phenotype and genotype have previously been reported [5]. The excised cornea was prepared for laser capture microdissection as previously described [4]. Thus, the tissue was fixed in 3.7% phosphate buffered neutral formaldehyde (formalin) followed by dehydration though graded alcohol solutions before being embedded in paraplast. The formalin-fixed and paraplast-embedded corneal tissue was cut in 5-µm-thick sections and three tissue sections were mounted on each glass microscope slide. Representative tissue sections were stained with Congo red and examined under polarized light according to standard procedures. Healthy corneal stroma used as the disease-free control was obtained from a 78-year-old female at the Department of Forensic Medicine, Aarhus University Hospital, Denmark. Corneal epithelial and endothelial cells were removed by scraping from the excised healthy cornea, and the stroma was cut into small pieces (about 1 mm^3^) with a scalpel. The human tissues were collected with the approval of the Internal Review Board of Duke University Medical Center and the Regional Committee for Scientific Medical Ethics in Aarhus, Denmark.

### Laser capture microdissection

An AutoPix Automated Laser Capture Microdissection (LCM) System (Arcturus Engineering, Inc., Mountain View, CA) was used to procure stromal amyloid deposits and periamyloid tissue from the diseased cornea on CapSure Macro LCM Caps (Arcturus Engineering, Inc.) as described elsewhere [4], except that the samples were not collected from the same tissue sections. Thus, from the 5-µm-thick tissue sections major stromal amyloid deposits and periamyloid tissue samples were selected manually and captured on separate CapSure Macro LCM Caps (Arcturus Engineering, Inc., Mountain View, CA) using the LCM system. The periamyloid tissue samples were procured from tissue sections containing fewer amyloid deposits. The LCM caps with procured tissue samples were saved at -20 °C for later mass spectrometry analyses. Only the most clearly defined captured amyloid deposits with little or no normal stroma attached were used for the analyses.

### Sample preparation and liquid chromatography-tandem mass spectrometry analyses

The laser capture microdissected corneal tissue samples were prepared for mass spectrometry analysis using the Liquid Tissue MS Protein Prep Kit (Expression Pathology Inc., Gaithersburg, MD) followed by the addition of RapiGest SF (Waters Corporation, Milford, MA; surfactant dissolved in 50 mM ammonium bicarbonate, pH 7.8) and trypsin (Sequencing Grade Modified Porcine Trypsin, Promega, Madison, WI) to generate peptides suitable for liquid chromatography-tandem mass spectrometry (LC-MS/MS) analysis as previously described [4]. Briefly, the LCM cap films with captured tissue samples were incubated with 20 µl of Liquid Tissue® Buffer (extraction buffer; Expression Pathology Inc., Gaithersburg, MD) at 95 °C for 90 min. The samples were then centrifuged for 1 min at 10,000 ×g and cooled on ice. Extracted proteins were then digested with 10 µg trypsin per reaction tube at 37 °C for 16 h. Subsequently, samples were incubated with 10 mM dithiothreitol at 95 °C for 5 min and centrifuged for 1 min at 10,000 ×g. Prior to mass spectrometry analysis, RapiGest SF dissolved in 50 mM ammonium bicarbonate, pH 7.8 was added to each sample to a concentration of 0.1% and trypsin was added to a concentration of 4% w/w to improve the protein digestion. The samples were then incubated at 37 °C overnight. For proteomic comparison, the pieces of healthy corneal stroma were also prepared using the Liquid Tissue MS Protein Prep Kit followed by digestion with trypsin in RapiGest SF. Peptides from 5 µl of the digestions were recovered using self-made C18-Stop and Go extraction tips (Stage-tips) [6], eluted with 20 µl of 70% acetonitrile, lyophilized, and the dried peptide samples were dissolved in 0.1% formic acid. Then the samples were analyzed by nanoLC-MS/MS using an EASY nLC system (Proxeon A/S, Odense, Denmark) coupled to a LTQ-Orbitrap XL mass spectrometer (Thermo Scientific, Bremen, Germany) equipped with a nano-electrospray ion source. Peptides were resolved on a 100 micron ID capillary column of 18 cm length packed with 3 micron Reprosil C18 by using a linear gradient of 0-34% B for 50 min (solvent B was 95% acetonitrile). The instrument threshold for fragmentation was set to 15.000 and the range was 300-1800. LC-MS/MS data were extracted from the Orbitrap raw files using Protein Discoverer with default settings.

### Identification and rough relative quantitation of proteins

The mass spectrometry data were processed, and the Swiss-Prot database was queried using an in-house Mascot search engine (Matrix Science, London, England; version 2.2.06) [7] with the same parameters and settings used previously [4]. Thus, the searches were performed at a peptide tolerance value of 10 ppm, a MS/MS tolerance value of 0.4 Da, no fixed modifications but methionine oxidation and proline hydroxylation as variable modifications, semiTrypsin as enzyme, 2 missed cleavages were allowed, and Electrospray ionization-Fourier Transform Ion Cyclotron Resonance (ESI-FTICR) mass spectrometer was selected as instrument in the Mascot program. In addition, “Require bold red” was selected to reduce the number of duplicate homologous protein identifications. Individual peptide scores lower than 20 were rejected, and only identifications with total protein scores higher than 40 were accepted (Appendices 1, 2, and 3). The MS/MS spectra of protein identifications based on less than three unique peptides were inspected manually, and were accepted only if they contained a clear sequence tag of at least three consecutive residues. Some of the identified proteins were regarded as contaminating common exogenous proteins (cexp), including keratin, type I cytoskeletal 9 (K1C9_HUMAN, P35527); keratin, type II cytoskeletal 1 (K2C1_HUMAN, P04264); keratin, type I cytoskeletal 10 (K1C10_HUMAN, P13645); keratin, type II cytoskeletal 2 epidermal (K22E_HUMAN, P35908); keratin, type II cytoskeletal 5 (K2C5_HUMAN, P13647); keratin, type I cytoskeletal 14 (K1C14_HUMAN, P02533); keratin, type I cytoskeletal 16 (K1C16_HUMAN, P08779); keratin, type I cytoskeletal 17 (K1C17_HUMAN, Q04695); dermcidin (DCD_HUMAN, P81605); keratin, type II cytoskeletal 6A (K2C6A_HUMAN, P02538); keratin, type II cytoskeletal 6B (K2C6B_HUMAN, P04259); keratin, type II cytoskeletal 6C (K2C6C_HUMAN, P48668) and hair keratins Ha1 (K1H1_HUMAN, Q15323); Ha3-I (KT33A_HUMAN, O76009); Ha3-II (KT33B_HUMAN, Q14525); Ha4 (KRT34_HUMAN, O76011); and Hb1 (KRT81_HUMAN, Q14533) [4,8-10] and according to the common Repository of Adventitious Proteins (cRAP list) [11]. Peptides from human trypsin (TRY3_HUMAN, P35030) identical to sequences in porcine trypsin (TRYP_PIG, P00761) were identified due to the addition of porcine trypsin and were, therefore, considered exogenous. Swiss-Prot accession names and numbers are indicated in brackets for each exogenous protein. The resulting non-contaminating endogenous proteins (enp) were listed according to their exponentially modified Protein Abundance Index (emPAI) values (emPAI^enp^) reflecting their relative abundances (Appendix 4). Since the microdissected stromal samples could not be collected from the same tissue sections, and the proteomic results strongly indicate that the protein composition in the periamyloid corneal tissue from the LCD type 1 variant is affected by the A546D mutation in TGFBIp, we did not perform emPAI-based CAPDan analyses on the data sets [4]. Instead, we compared the endogenous protein contents in molar fraction percentages (mol %) [12] calculated according to Equation 1. In contrast to the emPAI values, the mol % values are independent of the amount of injected protein. Proteins in the amyloid deposits and periamyloid tissue from the diseased cornea with a twofold increase in molar fraction compared to that in the healthy corneal stroma were defined as enriched.

(Equation 1)

$${\text{mol\%}}^{\text{enp}}=\frac{{\text{emPAI}}^{\text{enp}}}{\sum \left({\text{emPAI}}^{\text{enp}}\right)}100$$

### Categorization of proteins

The identified endogenous proteins were categorized in relation to their reported functional roles and cellular localization in other amyloidoses described in the current literature. The groups are as follows: Amyloidogenic proteins (proteins known to form amyloid fibers in other diseases); Non-fibrillar amyloid-associated proteins (proteins reported to associate with amyloid aggregates in other diseases, but are not themselves amyloidogenic in vivo); Structural extracellular matrix proteins (proteins reported to have structural roles in the extracellular matrix); Other extracellular matrix proteins (extracellular proteins with no structural role in the extracellular matrix); Structural intracellular proteins (proteins known to have a structural role in cells); Other intracellular proteins (intracellular proteins with no structural role); Membrane-bound proteins (proteins bound to the plasma membrane).

### Spectral counting for tryptic and semitryptic transforming growth factor beta induced protein peptides

For each MS-observable tryptic TGFBIp peptide (mass range 600–5400 Da) with no missed cleavages, the spectral count ratios for tryptic and semitryptic TGFBIp peptides were calculated as previously described [4]. Thus, the spectral count ratios for each of the MS-observable tryptic TGFBIp peptides (mass range 600 - 5400 Da) with no missed cleavages were calculated by dividing the count of spectra for the identified TGFBIp peptides with the total number of spectra for tryptic TGFBIp peptides or for semitryptic TGFBIp peptides and multiplying the result with 100. Tryptic peptides are generated exclusively by proteolysis at the C-terminal side of Lys or Arg residues, while semitryptic peptides are generated by proteolysis on the C-terminal side of a Lys or Arg residue in one terminus and another type of cleavage event at the other terminus. The spectral count ratios for tryptic peptides were used to compare peptide abundances, while spectral count ratios for semitryptic peptides were used to detect regions in TGFBIp with relatively higher levels of non-tryptic in vivo cleavages between the various corneal samples. Non-tryptic cleavages are any proteolytic cleavage events which occur not at the C-terminal side of Lys or Arg residues. Peptides with only non-tryptic cleavages were included in the group of semitryptic peptides. Since the trypsin preparation (Promega, Madison, WI) cleaves exclusively C-terminal to Arg or Lys residues and has shown no chymotrypsin activity [13], proteolytic events producing semitryptic peptides are likely the result of endogenous proteases. Error searches in Mascot were performed to identify the spectra of the mutant TGFBIp peptides.

## Results

### Microdissection of corneal amyloid deposits

**Figure 1:**
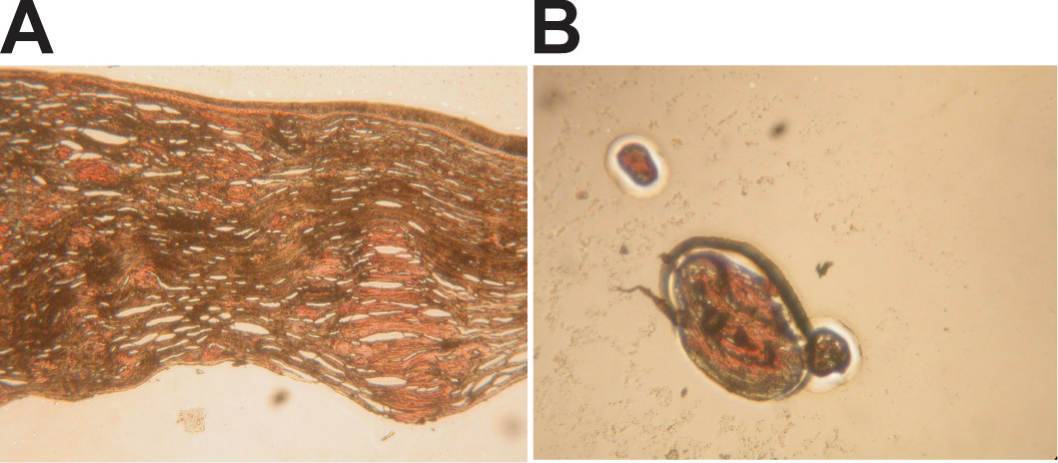
Capture of amyloid deposits from the cornea with lattice corneal dystrophy (LCD) type 1 variant by laser capture microdissection. Polymorphic amyloid deposits from a cornea with an LCD type 1 variant caused by the A546D mutation in the transforming growth factor beta induced (*TGFBI*) gene (**A**) were procured from tissue sections with laser capture microdissection (**B**). **A**: Prominent foci of amyloid were observed in formalin-fixed paraplast-embedded tissue sections of the grafted LCD type 1 variant cornea. **B**: Clearly defined amyloid deposits were easily procured from tissue sections and used for the proteomic analyses.

The tissue sections from the corneal stroma with the LCD type 1 variant contained prominent foci of amyloid (Figure 1). The amyloid material was accumulated in large irregular polygonal deposits interfused with the collagen layers of the stroma (Figure 1A). These major amyloid deposits were easily procured with laser capture microdissection (Figure 1B). Large regions of periamyloid tissue were captured from other sections of the tissue containing fewer amyloid deposits.

### Proteomic profiling of amyloid deposits, periamyloid tissue, and healthy corneal stroma

The proteomic profiling of the amyloid deposits resulted in 80 protein hits of which 16 proteins were considered contaminating common exogenous proteins (Appendices 1 and 4). The resulting 64 endogenous protein hits were listed according to the emPAI values, and showed that the ten most abundant proteins in the amyloid deposits were TGFBIp, type I collagen, serum amyloid P-component, keratocan, apolipoprotein A-IV, apolipoprotein A-I, clusterin, type VI collagen, serine protease HtrA1, and serum albumin (Table 1).

**Table 1:** Abundant proteins in the corneal amyloid deposits, periamyloid corneal tissue, and healthy corneal stroma.

**Amyloid deposits (LCD type 1 variant)**		**Periamyloid corneal tissue (LCD type 1 variant)**		**Healthy stroma (normal cornea)**	
Protein	mol %	Protein	mol %	Protein	mol %
**TGFBIp**	39.34	Type I collagen	22.33 (α-1) 14.07 (α-2)	TGFBIp	14.44
Type I collagen	21.16 (α-1) 6.47 (α-2)	TGFBIp	19.05	Type I collagen	12.75 (α-1) 8.49 (α-2)
**Serum amyloid P-component**	2.27	**Clusterin**	6.56	Decorin	6.53
Keratocan	2.27	**Apolipoprotein A-IV**	5.5	Keratocan	6.28
**Apolipoprotein A-IV**	1.66	**Phospholipase A2**	4.87	Type VI collagen	3.57 (α-1) 3.02 (α-3) 2.49 (α-2)
Apolipoprotein A-I	1.38	**Apolipoprotein D**	3.6	Serum albumin	3.49
**Clusterin**	1.35	**Serum amyloid P-component**	2.96	Apolipoprotein A-I	2.28
Type VI collagen	1.26 (α-3) 1.13 (α-1) 0.82 (α-2)	Serum albumin	2.12	Immunoglobulin gamma heavy chain (IgG)	1.81
**Serine protease HtrA1**	1.22	**Keratin 12**	2.12	Immunoglobulin kappa light chain (IgK)	1.77
Serum albumin	1.19	Keratocan	1.8	Lumican	1.41

The table shows the ten most abundant proteins in the amyloid deposits and periamyloid corneal tissue from the LCD type 1 variant, and healthy corneal stroma listed according to their mol % values. The different types of collagen are ranked according to the collagen chain with the highest emPAI-based molar fraction. The collagen chain species are indicated in brackets after the mol % values. Proteins shown in bold have at least a twofold increase in molar fraction compared to the healthy stroma.

From the periamyloid corneal tissue, we obtained 34 protein hits, of which 28 were endogenous proteins (Appendix 2 and Appendix 4). According to the emPAI values, the most abundant components of the periamyloid corneal tissue were type I collagen, TGFBIp, clusterin, apolipoprotein A-IV, phospholipase A2, apolipoprotein D, serum amyloid P-component, serum albumin, keratin 12, and keratocan (Table 1). The identification of clusterin, apolipoprotein A-IV, and serum amyloid P-component among the most abundant proteins in the periamyloid corneal tissue but not in the healthy corneal stroma suggests that the periamyloid tissue is not free of pathological effects. These proteins also accumulated in the amyloid from another patient with an LCD type 1 variant with a V624M mutation in *TGFBI* [4] and are known to associate with other misfolded proteins and amyloid deposits elsewhere in the body. We also identified serine protease HtrA1 in the periamyloid tissue from the cornea with LCD type 1 variant (Appendix 2 and Appendix 4). HtrA1 is normally associated with misfolded proteins and has previously been identified within corneal amyloid deposits in a patient with the V624M mutation in TGFBIp [4]. Altogether, this strongly suggests that the tissue surrounding the deposits in the LCD type 1 variant cornea (A546D) is involved in the pathologic process despite an apparent absence of amyloid according to light microscopy. Therefore, stroma from a healthy human cornea was analyzed and used as a disease-free reference for proteomic comparison.

Proteomic analysis of the healthy stroma disclosed 92 protein hits of which 91 were for endogenous proteins (Appendix 3 and Appendix 4). The most abundant proteins in the normal human corneal stroma were TGFBIp, type I collagen, decorin, keratocan, type VI collagen, serum albumin, apolipoprotein A-I, immunoglobulin gamma heavy chain (IgG), immunoglobulin kappa light chain (IgK), and lumican (Table 1).

A comparison of the emPAI-based molar fractions (mol %^enp^) of the most abundant proteins in the three different corneal specimens analyzed in this study disclosed that TGFBIp was most enriched in the amyloid deposits (Table 1). In addition, serum amyloid P-component (mol %^enp^ is 0.20 in healthy cornea), apolipoprotein A-IV (mol %^enp^ <0.02 in healthy cornea), clusterin (mol %^enp^ is 0.53 in healthy cornea), and serine protease HtrA1 (mol %^enp^ <0.02 in healthy cornea) were significantly enriched in the amyloid deposits (Table 1 and Appendix 4). In the periamyloid corneal tissue clusterin, apolipoprotein A-IV, phospholipase A2 (mol %^enp^ <0.02 in healthy cornea), apolipoprotein D (mol %^enp^ is 0.51 in healthy cornea), serum amyloid P-component, and keratin 12 (mol %^enp^ <0.02 in healthy cornea) were enriched compared to the healthy corneal stroma (Table 1 and Appendix 4).

### Categorization and description of proteins identified in amyloid deposits

All the endogenous proteins identified from the corneal TGFBIp amyloid deposits were categorized in groups according to the proteins’ functional roles and cellular localization in the current literature (Table 2). Of the total of 63 distinct proteins (Ig kappa light chain was identified with entries for the C-region and V-III regions), eight were amyloidogenic proteins, 14 were non-fibrillar amyloid-associated proteins, 14 were structural extracellular matrix proteins, eight were other extracellular matrix proteins, ten were structural intracellular proteins, eight were other intracellular proteins, and one was a membrane-bound protein.

**Table 2:** Categorization of proteins in groups.

**Amyloidogenic proteins**	**Non-fibrillar amyloid-associated proteins**	**Structural extracellular matrix proteins**	**Other extracellular matrix proteins**	**Structural intracellular proteins**	**Other intracellular proteins**	**Membrane-bound proteins**
TGFBIp	Serum amyloid P-component	Collagen α-1(I)	Serum albumin	Keratin 15	α-enolase	Desmoglein-1
Apolipoprotein A-IV	Clusterin	Collagen α-2(I)	S100-A7^a^	Keratin 13	Histone H2A type 1-A	
Apolipoprotein A-I	Serine protease HtrA1	Keratocan	Thioredoxin^a^	Keratin 3	ALDH3A1	
Ig kappa light chain, C and V-III	Apolipoprotein E	Collagen α-3(VI)	Angiopoietin-related protein 7	Keratin 12	52 kDa Ro protein	
Lysozyme C	Prostaglandin-H2 D-isomerase^a^	Collagen α-1(VI)	Cytokine-like protein 1	Keratin 75	Hornerin	
S100-A8	Complement component C9	Collagen α-2(VI)	Prolactin-inducible protein	Keratin 4	Desmoplakin	
S100-A9	Decorin	Lumican	PEDF	Keratin 71	Filaggrin-2	
Lactotransferrin	Apolipoprotein D	Collagen α-2(V)	Kallikrein-14	Keratin 1b	CAND1	
	Cystatin-A	Collagen α-1(III)		Keratin 23		
	Vitronectin	Collagen α-1(V)		Glial fibrillary acidic protein		
	Biglycan	Collagen α-1(VIII)				
	Glia-derived nexin	Prolargin				
	Complement component C3	Collagen α-4(IV)				
	Thrombospondin-4	Collagen α-1(XII)				
8 (13%)	14 (22%)	14 (22%)	8 (13%)	10 (16%)	8 (13%)	1 (2%)

The 63 distinct proteins identified in the amyloid deposits from the LCD type 1 variant with the A546D mutation in *TGFBI* are categorized according to their functional roles and cellular localization reported in current literature. The group with amyloidogenic proteins includes those that cause primary amyloidosis in other diseases. Non-fibrillar amyloid-associated proteins include those that are reported to bind or colocalize with amyloid deposits in other diseases, but are not themselves amyloidogenic in vivo. Structural extracellular matrix proteins include collagens and proteoglycans (leucine-rich repeat proteins), while other extracellular matrix proteins are categorized together. Structural intracellular proteins include intermediate filament proteins such as keratins, while other intracellular proteins are categorized together. Membrane-bound proteins are categorized in one group. The proteins in each group are listed according to relative abundances. The lowest row indicates the number of proteins in each group with the corresponding percentages in brackets. ^a^: the protein can be both extra- and intracellular.

### Amyloidogenic proteins

In addition to TGFBIp, the group of amyloidogenic proteins includes apolipoprotein A-IV forming amyloid in systemic amyloidosis [14,15] and is known to specifically accumulate in corneal TGFBIp amyloid deposits [4], apolipoprotein A-I known from apolipoprotein A-I amyloidosis [16], Ig kappa light chain forming amyloid in immunoglobulin light chain amyloidosis (composed of the Ig light chain constant or variable region) [17-20], lysozyme C causing lysozyme amyloidosis [21,22], proteins S100-A8 and A9 reported to form amyloid in corpora amylacea in prostate glands [23], and lactotransferrin (lactoferrin) forming amyloid in the cornea [24-26].

### Non-fibrillar amyloid-associated proteins

Among these proteins, serum amyloid-P component, clusterin, apolipoprotein E, and vitronectin are known to interact with several amyloid aggregates [4,27-31]. In addition, serine protease HtrA1 [32], prostaglandin-H2 D-isomerase (also termed lipocalin-type prostaglandin D synthase/β-trace) [33], apolipoprotein D [34], thrombospondin-4 [35], cystatin-A [36], glia-derived nexin [37], complement factors C3 and C9 [38-41], and the proteoglycan decorin [42] colocalize with β-amyloid plaques in Alzheimer disease. Furthermore, decorin and the other proteoglycan biglycan have also been reported to associate with renal amyloid deposits [43,44].

### Serine proteases and inhibitors

In addition to serine protease HtrA1, which we have recently reported to associate with corneal TGFBIp amyloid in an LCD type 1 variant caused by the V624M mutation in TGFBIp [4], we identified the extracellular serine protease kallikrein-14 (kallikrein-related peptidase 14) [45,46] in the corneal amyloid samples linked to the A546D mutation in *TGFBI*. Human kallikreins are implicated in normal physiological processes as well as various diseases, including arthritis, cancer, and neurodegenerative diseases. Interestingly, we identified a serine protease inhibitor (serpin), glia-derived nexin (also termed protease-nexin 1), in the amyloid deposits (Table 2). Glia-derived nexin has been found to associate with neuritic plaques in Alzheimer disease [37] and is known to inhibit several serine proteases that cleave at basic residues (the P1 position) including thrombin, urokinase plasminogen activator (uPA) [47], trypsin, plasmin, Factor XIIa, and plasma kallikrein [48,49].

### Differential proteolytic processing and accumulation of transforming growth factor beta induced protein fragments in amyloid and periamyloid corneal samples

**Figure 2:**
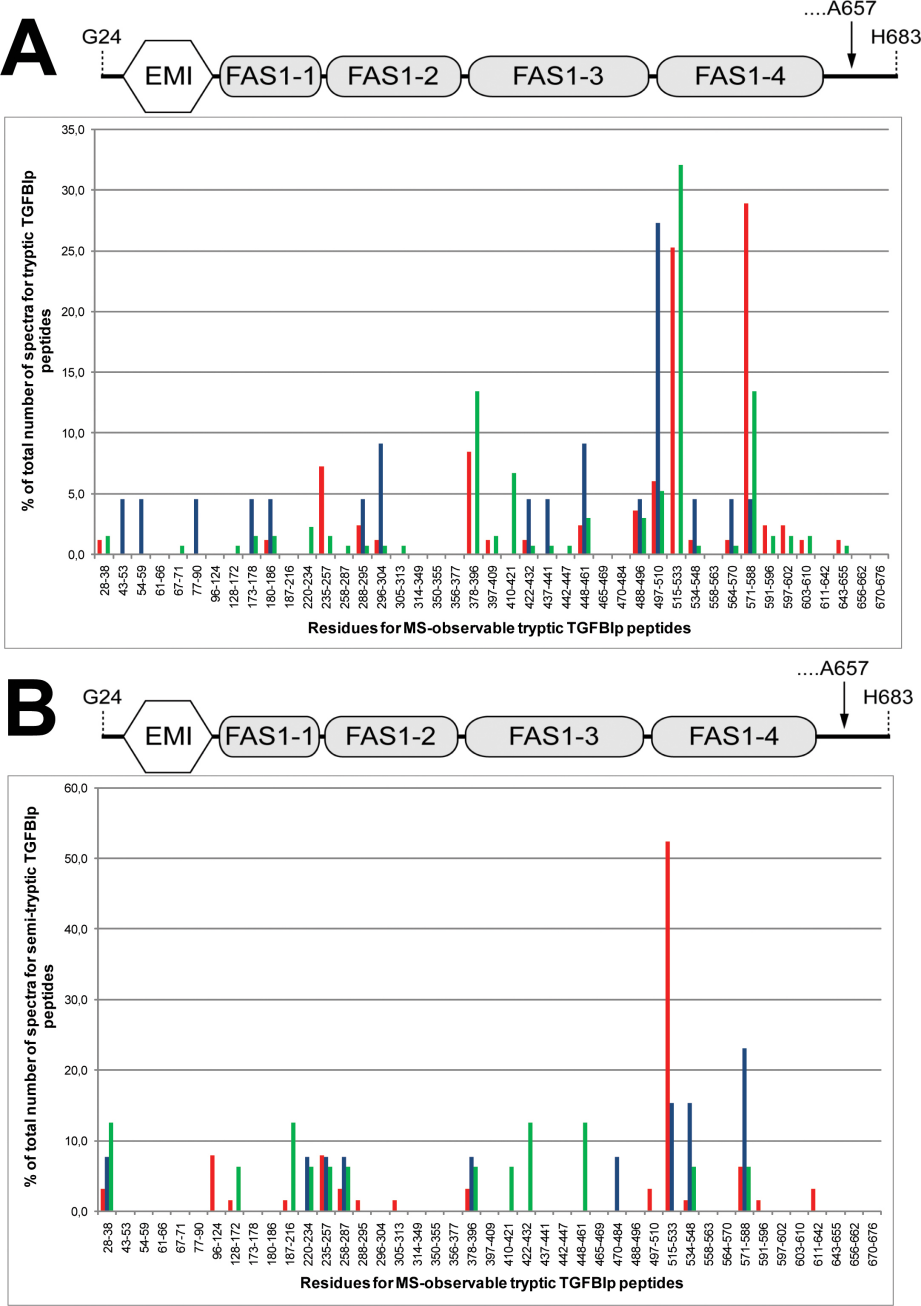
Spectral count ratios for tryptic and semitryptic transforming growth factor beta induced protein (TGFBIp) peptides. Spectral count ratios for tryptic TGFBIp peptides (**A**) and semitryptic TGFBIp peptides (**B**) detected in the amyloid deposits (red bars), periamyloid corneal tissue (blue bars), and healthy stroma (green bars). The spectral count ratios are plotted for each mass spectrometry (MS)-observable tryptic TGFBIp peptide with no missed cleavages. In the amyloid deposits, the highest spectral count ratios for tryptic peptides were observed for Y571-R588 and F515-R533 located in the fourth fasciclin 1 (FAS1-4) domain. Other tryptic TGFBIp peptides with relatively high spectral count ratios (V235-R257 and T378-R396) were located at the inter-domain regions. For the semitryptic peptides, the highest spectral count ratio was for peptide F515-R533. In the periamyloid corneal tissue, the highest spectral count ratio for tryptic TGFBIp peptides was observed for peptide V497-K510. For the semitryptic TGFBIp peptides in the periamyloid tissue, the highest spectral count ratio was for residues Y571-R588. No tryptic or semitryptic peptides from the C-terminal region covering residues S591-K676 of TGFBIp were detected in the periamyloid corneal tissue. The schematic drawing above the bar charts shows the domain structure of TGFBIp to illustrate the locations of the peptides. The structure includes the N-terminal EMILIN domain (EMI, residues 45–99) and the four consecutive fasciclin 1 (FAS1) domains (FAS1–1: residues 103–235; FAS1–2: residues 242–371; FAS1–3: residues 376–498; and FAS1–4: residues 505–632). The arrow indicates that the major isoform of TGFBIp in the human cornea is cleaved at the C-terminus of residue A657.

As expected, TGFBIp is the most abundant protein in the amyloid deposits in the cornea with an A546D mutation in *TGFBI* (Table 1). To further investigate the accumulation and proteolytic processing of TGFBIp in the diseased cornea, we compared the spectral count ratios for tryptic and semitryptic TGFBIp peptides among the amyloid deposits, periamyloid tissue, and healthy corneal stroma (Figure 2). In the amyloid samples, we detected 83 and 63 spectral counts for tryptic and semitryptic TGFBIp peptides, respectively. In the periamyloid corneal tissue, we detected 22 and 13 spectral counts for tryptic and semitryptic TGFBIp peptides, respectively, while 134 and only 16 spectral counts were observed for tryptic and semitryptic TGFBIp peptides from the healthy corneal stroma, respectively. The high ratios of the spectral counts for semitryptic TGFBIp peptides compared to tryptic TGFBIp peptides in the LCD type 1 samples suggests that TGFBIp is processed significantly more proteolytically in the dystrophic corneas compared to the healthy corneal tissue.

### Amyloid deposits

In the amyloid deposits, tryptic peptides from all parts of TGFBIp were detected (Figure 2A, red bars). However, the identified tryptic peptides were mainly derived from the C-terminal part of TGFBIp, and the highest spectral count ratios for tryptic peptides were observed for peptides Y571-R588 and F515-R533 located in the middle and beginning of the FAS1–4 domain, respectively (Figure 2A). Other tryptic TGFBIp peptides with relatively high spectral count ratios were V235-R257 and T378-R396 located at the inter-domain regions of the first and second FAS1 domains and the second and third FAS1 domains, respectively. For the semitryptic peptides, more than 50% of the total spectral counts were from the F515-R533 region, making this the peptide with the highest spectral count ratio (Figure 2B, red bars).

### Periamyloid corneal tissue

The amyloid-deficient tissue surrounding areas of amyloid deposition contained tryptic peptides from all parts of TGFBIp except the C-terminal sequence S591-K676 (Figure 2A, blue bars). The highest spectral count ratio in this corneal tissue was for tryptic peptide V497-K510 located at the inter-domain region of the FAS1–3 and FAS1–4 domains. The semitryptic TGFBIp peptides identified in the periamyloid samples were mainly from the FAS1–4 domain. Thus, the highest spectral count ratio for the semitryptic TGFBIp peptides in the periamyloid corneal tissue was for region Y571-R588 (Figure 2B, blue bars).

### Healthy cornea

In the healthy human cornea, tryptic peptides from all parts of TGFBIp were detected, but the highest spectral count ratio was observed for region F515-R533 (Figure 2A, green bars). For the semitryptic TGFBIp peptides, no region had a predominant spectral count ratio (Figure 2B, green bars).

Taken together, these findings suggest that a C-terminal fragment of TGFBIp containing residues Y571-R588 was relatively more abundant in the amyloid deposits than in the corneal stroma adjacent to the amyloid in the LCD type 1 variant as well as in the healthy corneal stroma. In contrast, TGFBIp isoforms that are cleaved in the Y571-R588 sequence and therefore lacking the C-terminal part (no tryptic or semitryptic peptides are detected from the region S591-K676) were abundant in the periamyloid corneal tissue from the diseased cornea (Figure 2, blue bars).

### Non-tryptic cleavages of amyloid transforming growth factor beta induced protein are clustered in the beginning of the fourth fasciclin 1 domain

**Figure 3:**
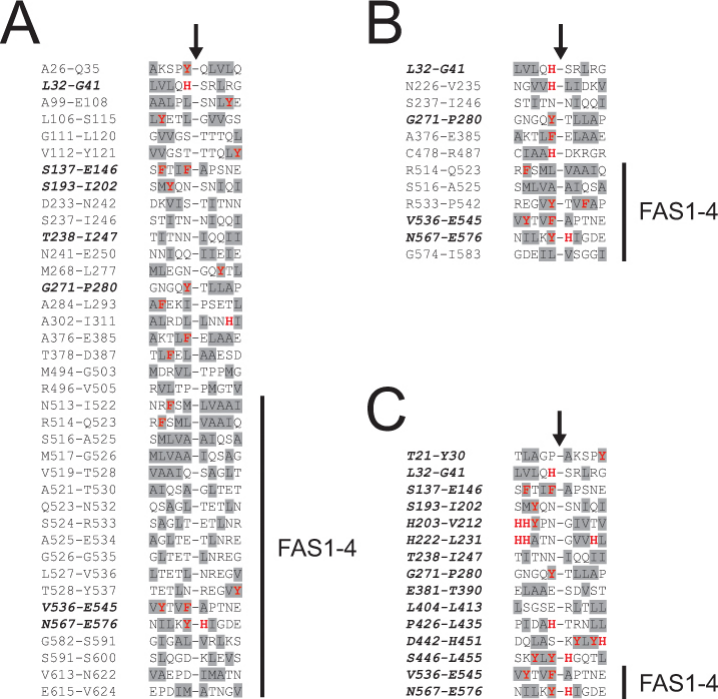
Alignment of the in vivo cleavage sites in transforming growth factor beta induced protein (TGFBIp) from the lattice corneal dystrophy (LCD) type 1 variant and healthy corneas. The sequences of the non-tryptic cleavage sites in TGFBIp from the amyloid deposits (**A**), periamyloid corneal tissue (**B**), and the healthy corneal stroma (**C**) were aligned (from position −5 to +5). Residues with aromatic side chains (F, Y, W, H) and hydrophobic side chains (A, I, L, M, F, W, Y, V) are marked with red bold letters and gray background, respectively. The residue numbers in TGFBIp of the aligned sequences are indicated, and the black arrows show the cleavage sites. The position of the amyloidogenic fourth fasciclin 1 (FAS1-4) domain (residues 505–632) is indicated in the alignments. (**A**) The TGFBIp FAS1–4 domain from the amyloid deposits is predominantly cleaved at the C-termini of the hydrophobic residues leucine and alanine. (**B**) All proteolytic cleavages in the TGFBIp FAS1–4 domain from the periamyloid tissue from the LCD type 1 variant cornea occur at the C-termini of the aromatic side chains (F, Y) and the hydrophobic side chains (A, L). (**C**) The two in vivo cleavages detected in the TGFBIp FAS1–4 domain from the healthy cornea were at the C-termini of the aromatic residues phenylalanine and tyrosine. Sequences shown in bold italics indicate cleavage sites, which are also identified in the healthy cornea.

To further study the in vivo processing of TGFBIp in the amyloid and the periamyloid portions of the pathologic human corneal tissue, the non-tryptic cleavage sites were mapped and compared with those identified from the healthy cornea (Appendix 5). In total, 38 and 12 distinct non-tryptic cleavage sites were identified in TGFBIp from the amyloid and periamyloid corneal tissue, respectively. From the healthy corneal stroma, 15 different non-tryptic cleavage sites were identified in TGFBIp. Comparison of the proteolytic events revealed that 31 of the 38 TGFBIp cleavage sites (82%) from the amyloid deposits and eight of the 12 cleavage sites (67%) from the periamyloid tissue were not detected in the healthy cornea tissue, suggesting that altered proteolytic events occur in LCD type 1 (Appendix 5). Previous studies have shown that TGFBIp from normal human corneas is proteolytically processed [9,50], but except for the cleavage at the C-terminus of A657 in mature TGFBIp [51], the specific cleavage sites have remained unknown. The cleavage sites in normal TGFBIp from the healthy cornea were distributed throughout the TGFBIp sequence, while those from the amyloid deposits were more clustered (Appendix 5A). Thus, many of the cleavage sites in TGFBIp from the amyloid deposits were found in the sequence F515-R533 at the beginning of the FAS1–4 domain and in close proximity to the mutated residue A546 (Appendix 5A). Likewise, half of the cleavage sites in TGFBIp from the periamyloid samples were located in the FAS1–4 domain and flanked the mutation (Appendix 5B). To further investigate the proteolytic cleavage sites observed in the LCD type 1 and healthy corneas, the sequences were compared (Figure 3). Alignment analyses revealed that in all samples (amyloid deposits, periamyloid corneal tissue, and healthy corneal stroma) the proteolytic cleavages in the amyloidogenic FAS1–4 domain occurred predominantly or exclusively at the C-termini of aromatic side chains (F, Y) and hydrophobic side chains (A, L, M). However, some of the cleavages in the FAS1–4 domain of TGFBIp from the amyloid deposits were at the C-termini of T, D, E, Q, and N (Figure 3A). Specifically, all six cleaved sites in the FAS1–4 domain of TGFBIp from the periamyloid tissue were at the C-termini of aromatic residues (F, Y) or hydrophobic side chains (A, L; Figure 3B). The cleavages at the C-termini of residues F540 and Y571 in the TGFBIp FAS1–4 domain from the healthy cornea (Figure 3C) were also present in tissue with LCD type 1 (Figure 3A,B) indicating that these specific proteolytic events are part of the normal processing or turnover of TGFBIp in the human cornea.

## Discussion

The biochemical processes that occur in *TGFBI*-linked cornea amyloidosis were investigated with in-depth proteomic analyses of the amyloid deposits and periamyloid corneal tissue laser captured from a human cornea with an LCD type 1 variant caused by a heterozygous A546D mutation in *TGFBI*. The results were compared with the proteomic profiling of a healthy corneal stroma sample. Together with recent results obtained from another variant of LCD type 1 caused by the homozygous mutation V624M in *TGFBI* [4], the present proteomic profiling of the amyloid deposits suggests that LCD type 1 variants caused by mutations in the FAS1–4 domain of TGFBIp lead to the accumulation and deposition of the same TGFBIp fragment(s) and proteins in the cornea. Significantly, the study reveals that serine protease HtrA1 is a major component of the corneal amyloid deposits and proteolytic cleavages in TGFBIp from the diseased cornea are in accordance with the activity of serine protease HtrA1.

### Proteins enriched in the transforming growth factor beta induced protein amyloid are associated with protein misfolding diseases

It is particularly noteworthy that most of the major constituents in the amyloid deposits from the LCD type 1 variant cornea with the A546D mutation in *TGFBI* (Table 1) are identical to those identified in another variant of this disease due to the V624M mutation in *TGFBI* [4]. However, the serine protease HtrA1 is relatively much more abundant, and apolipoprotein E is less abundant in the amyloid samples from the A546D mutant cornea. The calculated emPAI-based molar fractions of the proteins in the corneal samples suggest that TGFBIp is enriched in the TGFBIp amyloid deposits compared to the periamyloid corneal tissue and healthy corneal samples (Appendix 4 and Table 1). Based on the relative abundances, we conclude that TGFBIp, serum amyloid P-component, apolipoprotein A-IV, clusterin, and serine protease HtrA1 are enriched in the amyloid deposits while clusterin, apolipoprotein A-IV, phospholipase A2, apolipoprotein D, serum amyloid P-component, and keratin 12 are enriched in the periamyloid samples compared to the healthy corneal stroma (Table 1). All the major enriched proteins in the TGFBIp amyloid are known to be abundant in other protein misfolding diseases where the proteins either form amyloid or associate with the amyloid deposits (Table 2). Thus, our results suggest that the same components accumulate in TGFBIp amyloid in LCD type 1 when caused by either the A546D or V624M mutation in *TGFBI* [4], but the relative protein abundances vary between the two different specimens. In addition, the A546D mutant TGFBIp not only affects the tissue regions containing the amyloid deposits but also lead to altered protein composition in the periamyloid corneal tissue.

### Codeposition of well-known amyloidogenic proteins in the corneal transforming growth factor beta induced protein amyloid deposits suggests heterologous amyloid seeding in vivo

The in-depth analyses of the identified proteins in the amyloid deposits revealed that many of the proteins are well-known amyloidogenic proteins or are known to associate with amyloid in other amyloidoses (Table 2). Previously, the amyloidogenic protein apolipoprotein A-IV was shown to specifically accumulate in the corneal amyloid deposits from the LCD type 1 variant caused by the V624M mutation in *TGFBI* [4]. The presence of several other amyloidogenic proteins in the deposits could indicate that these minor to moderate amyloid constituents are incorporated in the growing TGFBIp amyloid fibrils or fibrillate in parallel as they recognize the characteristic beta-sheet structures of the amyloids. Only a few studies have previously reported colocalization of more amyloidogenic proteins in the amyloid deposits including transthyretin and apolipoprotein A-I [52] and transthyretin and immunoglobulin kappa light chain [53]. In addition, studies have reported evidence of the existence of heterologous amyloid seeding and fibrils [54-56]. Thus, the colocalization of several amyloid-forming proteins including apolipoprotein A-IV, apolipoprotein A-I, Ig kappa chain, lysozyme C, proteins S100-A8 and A9, and lactoferrin with the corneal TGFBIp amyloid deposits indicates that these proteins are constituents of the amyloid fibrils.

### A C-terminal transforming growth factor beta induced protein fragment containing residues Y571-R588 accumulates in the amyloid deposits

Comparison of the spectral counts for tryptic TGFBIp peptides from the healthy cornea, periamyloid corneal tissue, and amyloid deposits suggests that a fragment from the FAS1–4 domain containing residues Y571-R588 accumulates in the amyloid deposits in the LCD type 1 variant caused by the A546D mutation in *TGFBI* (Figure 2A). In accordance with this, most of the non-tryptic cleavage sites were observed in the beginning of the FAS1–4 domain, specifically within residues F515-R533 (Figure 2B). The Y571-R588 peptide of TGFBIp has also been found to specifically accumulate in the corneal amyloid in the LCD type 1 variant due to the V624M mutation in *TGFBI* [4], thus supporting the idea that this region is responsible for amyloidosis in LCDs linked to mutations in the FAS1–4 domain of TGFBIp. However, the observed enrichment of the Y571-R588 in the amyloid deposits does not exclude the contribution of other TGFBIp regions in the formation of corneal deposits. Thus, a relatively high spectral count ratio was also observed for peptide F515-R533 (Figure 2A). This region contains a putative amyloidogenic motif (residues F515-N532) reported previously [57] and, therefore, may be involved in the amyloid formation in the variant of LCD type 1 caused by the A546D mutation in *TGFBI*. However, further studies are required to determine the specific roles of these FAS1–4 domain regions in the pathobiology of TGFBIp-linked corneal amyloidosis.

Comparison of the spectral count ratios of TGFBIp peptides for the different samples suggested that TGFBIp isoforms lacking the C-terminal region are predominant in the periamyloid corneal stroma (Figure 2). In accordance with this, we observed a relatively low spectral count ratio for tryptic peptide Y571-R588 and a higher ratio for semitryptic TGFBIp peptides in region Y571-R588 from the periamyloid corneal stroma (Figure 2). Specifically, these non-tryptic cleavages in region Y571-R588 of TGFBIp from the periamyloid corneal stroma are at the C-terminal side of Y571 and L578 (Figure 3B). In a previous study, we suggested that the physiologically relevant amyloidogenic-prone sequence (amyloid core region) in the fourth FAS1 domain of TGFBIp is within sequence 571-YHIGDEILVSGGIGALVR-588. Therefore, proteolytic processing and elimination of this sequence may prevent the formation of amyloid in vivo perhaps explaining the absence of amyloid in the periamyloid corneal tissue of the diseased cornea. In a recent study, we showed that the amyloidogenic A546T mutation destabilizes the FAS1–4 domain as well as intact TGFBIp and increases the propensity of amyloid fibril formation [3]. Based on those results, it seems reasonable to believe that the A546D substitution in TGFBIp will also destabilize the FAS1–4 domain, which could lead to exposure of the amyloidogenic-prone sequence in the FAS1–4 domain and amyloid fibril formation. In vivo cleavages of the Y571-R588 sequence will likely alter the domain arrangement of TGFBIp. Thus, the high spectral count ratio for tryptic TGFBIp peptide V497-K510 (Figure 2A) in the periamyloid corneal tissue may be explained by an altered domain arrangement of TGFBIp making it easier for trypsin to cleave at the inter-domain region of the FAS1–3 and FAS1–4 domains [4].

### Serine protease HtrA1, kallikrein-14, and inhibitor glia-derived nexin are associated with the corneal transforming growth factor beta induced protein amyloid

Serine protease HtrA1 accumulated in the amyloid deposits (Table 1) and was also found in the periamyloid corneal tissue of the LCD cornea (Appendix 2), which makes serine protease HtrA1 a highly potential candidate responsible for the proteolytic processing of TGFBIp. HtrA1 has previously been shown to colocalize with the corneal amyloid associated with the V624M mutation in *TGFBI* [4] and with β-amyloid plaques in Alzheimer disease [32]. In addition, recent results have shown that HtrA1 is able to degrade tau protein aggregates [58]. Out of the 18 non-tryptic cleavage sites in TGFBIp identified from the amyloid deposits captured from a patient with *TGFBI* mutation V624M [4], 14 were also found in TGFBIp amyloid in the present study. A screening of ligand binding performed by Murwantoko and colleagues revealed that HtrA1 recognizes protein C-terminal ends with consensus sequence Phi-X-Phi-[V/L/F/A]-COOH (where Phi is a hydrophobic/non-polar amino acid and X is any amino acid) [59], while Truebestein et al. showed that this protease cleaves substrates with L, V, A, T, M, S, I, Q, and E in the P1 position with a decreasing preference [60]. In addition, reported native substrate cleavages at the C-termini of V, Q, N, L, G, and H in amyloid precursor protein fragments [32] and V in aggrecan [61] show that the protease has no immense preference for specific residues at the P1 position but rather recognizes and cleaves unstructured hydrophobic sequences. With a few exceptions, all the cleavage sites we identified in the FAS1–4 domain of TGFBIp (Figure 3) are consistent with the reported ligand binding and proteolytic activity of HtrA1. Specifically, all cleavages in the “proteolytic hotspot” (F515-R533) in TGFBIp from the amyloid deposits (Figure 2B) occur at the C-termini of residues L, A, T, M, Q, E, and N (Appendix 5A and Figure 3A). Therefore, the cleavage sites in TGFBIp together with the identification of HtrA1 as a major constituent of the corneal amyloid suggest that this protease is responsible for the proteolytic processing of aggregated amyloidogenic TGFBIp. Likewise, HtrA1 may also be responsible for the proteolytic processing of destabilized mutant TGFBIp in the periamyloid regions of the diseased cornea. Based on our present results and those obtained from another variant of LCD type 1 caused by homozygous mutation V624M in *TGFBI* [4], we suggest that serine protease HtrA1 cleaves partly unfolded mutant TGFBIp in the soluble state (periamyloid tissue) but also tries to remove the insoluble TGFBIp amyloid aggregates, which explains the accumulation of serine protease HtrA1 in the deposits. However, we also identified small amounts of serine protease kallikrein-14 in the amyloid deposits. Kallikrein-14 cleaves with high selectivity at the C-terminal site of arginine and lysine residues [62], but cleavage after tyrosine has also been reported [63]. Thus, kallikrein-14 may be responsible for the few identified cleavages at the C-terminal site of tyrosine residues in TGFBIp (Figure 3). However, further investigations are required to confirm that amyloidogenic mutant TGFBIp, and specifically the FAS1–4 domain, is a substrate for serine proteases HtrA1 and kallikrein-14 under physiological conditions in vitro. The fact that the serpin glia-derived nexin (protease-nexin 1) was found in the amyloid deposits suggests that it is involved in regulating the proteolytic processing of the TGFBIp amyloid and makes glia-derived nexin a good candidate for the physiologically relevant inhibitor of one of the amyloid-associated serine proteases in the cornea and probably in other tissues. Glia-derived nexin inhibits serine proteases cleaving at basic residues and, therefore, most likely regulates the proteolytic activity of kallikrein-14 in vivo.

### Composition of amyloid deposits suggest common aggregation and codeposition mechanisms for lattice corneal dystrophies associated with transforming growth factor beta induced protein mutations in the fourth fasciclin 1 domain

Based on these results, we conclude that a C-terminal TGFBIp fragment encompassing residues Y571-R588 from the fourth FAS1 domain, serum amyloid P-component, apolipoprotein A-IV, clusterin, and serine protease HtrA1 accumulate in the corneal amyloid deposits from the LCD type 1 variant associated with the A546D mutation in *TGFBI*. Together with our previous findings, the present study suggests that TGFBIp-linked corneal amyloidoses caused by FAS1–4 domain mutations located before and after residues Y571-R588 lead to the accumulation of the same C-terminal fragment containing the Y571-R588 region. Therefore, we propose that sequence 571-YHIGDEILVSGGIGALVR-588 contains the amyloid core of the fourth FAS1 domain of TGFBIp. However, we cannot exclude that other regions of TGFBIp may contribute in forming corneal *TGFBI*-linked amyloid deposits. In addition, the proteomic profile and identified in vivo cleavages in TGFBIp of the amyloid deposits from the LCD type 1 variant due to the A546D mutation in *TGFBI* are in good accordance with our recent results on the composition and proteolytic processing of amyloid associated with the V624M mutation in *TGFBI* [4]. Thus, our results suggest common mechanisms for aggregating and codepositing proteins in the amyloid deposits associated with mutations in the fourth fasciclin 1 domain of TGFBIp.

## Appendix 1. Proteins identified in amyloid deposits from LCD type 1 cornea with A546D mutation in *TGFBI*.

The protein hits are numbered (prot_hit_num) according to the Protein Score (prot_score). In addition, the following information are available in the data set: protein accession name in the Swiss-Prot database (prot_acc), description (prot_desc), protein mass (prot_mass), percentage of protein sequence covered by assigned peptide matches (prot_cover), the assigned MS/MS spectrum number (pep_query), rank of peptide match (pep_rank), indication of first time (1) or not first time (0) the peptide is matched to a protein in the data set (pep_isbold), observed m/z value (pep_exp_mz), observed relative molecular mass (pep_exp_mr), observed charge state (pep_exp_z), calculated relative molecular mass (pep_calc_mr), pep_calc_mr - pep_exp_mr (pep_delta), first and last residue numbers in the matched peptide (pep_start and pep_end), number of missed trypsin cleavage sites (pep_miss), peptide match score (pep_score), peptide match expectation value (pep_expect), the sequence of the peptide (pep_seq) and the residue before (pep_res_before) and the residue after (pep_res_after) the peptide sequence in the protein, variable modifications (methionine oxidation and proline hydroxylation) in the peptide (pep_var_mod) and their position in the peptide sequence (pep_var_mod_pos), and emPAI value for the protein hit (emPAI). To access the data, click or select the words “Appendix 1.”

## Appendix 2. Proteins identified in periamyloid corneal tissue from LCD type 1 cornea with A546D mutation in *TGFBI*.

The protein hits are numbered (prot_hit_num) according to the Protein Score (prot_score). In addition, the following information are available in the data set: protein accession name in the Swiss-Prot database (prot_acc), description (prot_desc), protein mass (prot_mass), percentage of protein sequence covered by assigned peptide matches (prot_cover), the assigned MS/MS spectrum number (pep_query), rank of peptide match (pep_rank), indication of first time (1) or not first time (0) the peptide is matched to a protein in the data set (pep_isbold), observed m/z value (pep_exp_mz), observed relative molecular mass (pep_exp_mr), observed charge state (pep_exp_z), calculated relative molecular mass (pep_calc_mr), pep_calc_mr - pep_exp_mr (pep_delta), first and last residue numbers in the matched peptide (pep_start and pep_end), number of missed trypsin cleavage sites (pep_miss), peptide match score (pep_score), peptide match expectation value (pep_expect), the sequence of the peptide (pep_seq) and the residue before (pep_res_before) and the residue after (pep_res_after) the peptide sequence in the protein, variable modifications (methionine oxidation and proline hydroxylation) in the peptide (pep_var_mod) and their position in the peptide sequence (pep_var_mod_pos), and emPAI value for the protein hit (emPAI). To access the data, click or select the words “Appendix 2.”

## Appendix 3. Proteins identified in healthy stroma from a normal human cornea.

The protein hits are numbered (prot_hit_num) according to the Protein Score (prot_score). In addition, the following information are available in the data set: protein accession name in the Swiss-Prot database (prot_acc), description (prot_desc), protein mass (prot_mass), percentage of protein sequence covered by assigned peptide matches (prot_cover), the assigned MS/MS spectrum number (pep_query), rank of peptide match (pep_rank), indication of first time (1) or not first time (0) the peptide is matched to a protein in the data set (pep_isbold), observed m/z value (pep_exp_mz), observed relative molecular mass (pep_exp_mr), observed charge state (pep_exp_z), calculated relative molecular mass (pep_calc_mr), pep_calc_mr - pep_exp_mr (pep_delta), first and last residue numbers in the matched peptide (pep_start and pep_end), number of missed trypsin cleavage sites (pep_miss), peptide match score (pep_score), peptide match expectation value (pep_expect), the sequence of the peptide (pep_seq) and the residue before (pep_res_before) and the residue after (pep_res_after) the peptide sequence in the protein, variable modifications (methionine oxidation and proline hydroxylation) in the peptide (pep_var_mod) and their position in the peptide sequence (pep_var_mod_pos), and emPAI value for the protein hit (emPAI). To access the data, click or select the words “Appendix 3.”

## Appendix 4. Proteins identified in corneal amyloid deposits, periamyloid corneal tissue, and healthy corneal stroma listed according to emPAI.

The identified proteins in corneal amyloid deposits, periamyloid corneal tissue, and healthy corneal stroma are listed according to emPAI values. The description (prot_desc) and protein accession name in the Swiss-Prot database (prot_acc) are available for each protein. The emPAI values are indicated for the endogenous proteins (emPAI^enp^) and the common exogenous proteins (emPAI^cexp^). Trypsin-3 (marked in bold) is classified as exogenous protein (emPAI^cexp^ (trypsin)). The molar fractions in percentages for the endogenous proteins (mol %^enp^) are calculated. To access the data, click or select the words “Appendix 4.”

## Appendix 5. Locations of the non-tryptic cleavage sites in transforming growth factor beta induced protein (TGFBIp) from corneal amyloid deposits, periamyloid corneal tissue, and healthy cornea.

Identified N- and C-termini which are not the result of trypsin digestion are indicated on the sequence of TGFBIp with the heterozygous A546D mutation indicated with a red D-letter above the sequence for the amyloid deposits (**A**) and periamyloid corneal tissue (**B**) from the LCD type 1 variant cornea. The positions of the cleavage sites in TGFBIp from the diseased cornea are compared with those found in the healthy cornea. Black and white arrows represent proteolytic cleavages identified only in the LCD type 1 variant corneal sample and healthy cornea, respectively. Gray arrows represent proteolytic cleavages identified in both the LCD type 1 variant corneal sample and healthy cornea. The underlined residue at each cleavage site indicates the terminus of the identified semitryptic peptide. The numbers above the arrows are the spectral counts of the peptide(s) with the proteolytic cleavage observed from the diseased cornea (*upper number*) and the healthy cornea (*lower number*). The colored bars below the sequences indicate the positions of the four FAS1 domains (FAS1–1: residues 103–235 (green); FAS1–2: residues 242–371 (red); FAS1–3: residues 376–498 (blue); and FAS1–4: residues 505–632 (yellow)). The cleavage sites detected only in TGFBIp from the healthy cornea are dispersed throughout the sequence. (**A**) Many of the cleavage sites in TGFBIp from the amyloid deposits are clustered in region F515-R533 positioned close to the mutated residue A546. (**B**) Half of the cleavage sites in TGFBIp from the periamyloid corneal sample are also located in the FAS1–4 domain in close proximity to the mutated residue A546. To access the data, click or select the words “Appendix 5.”
